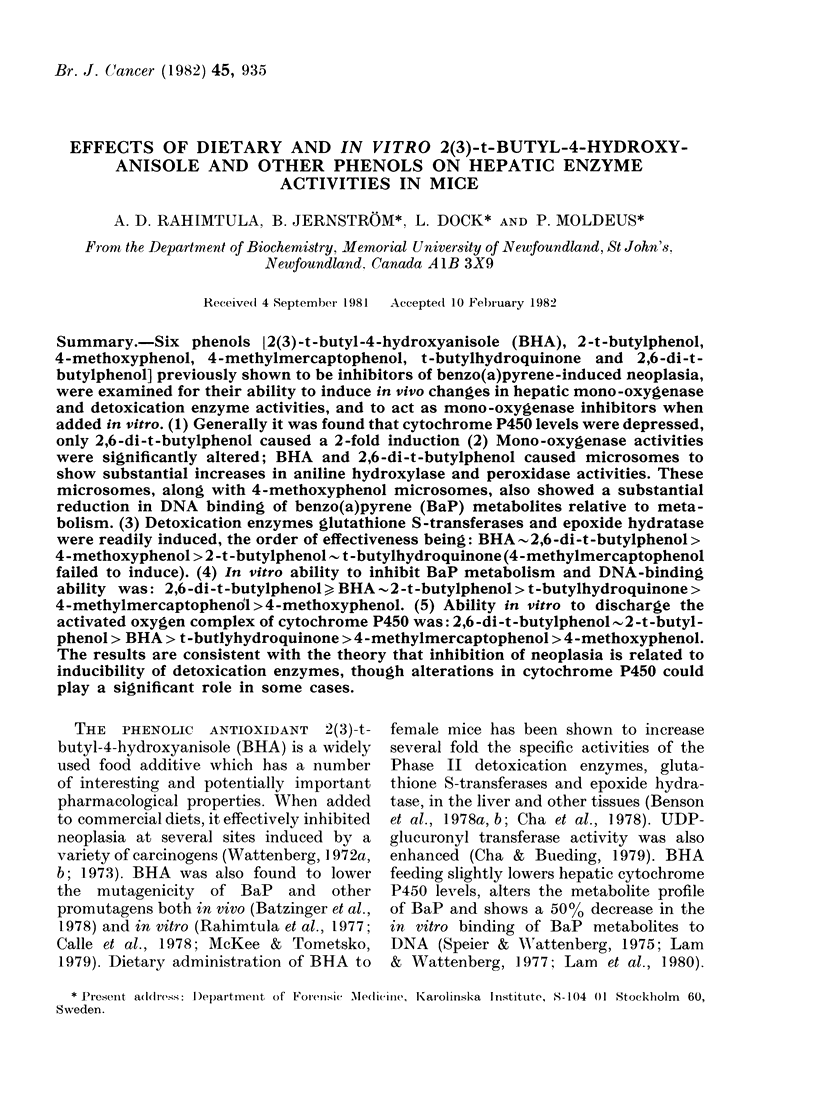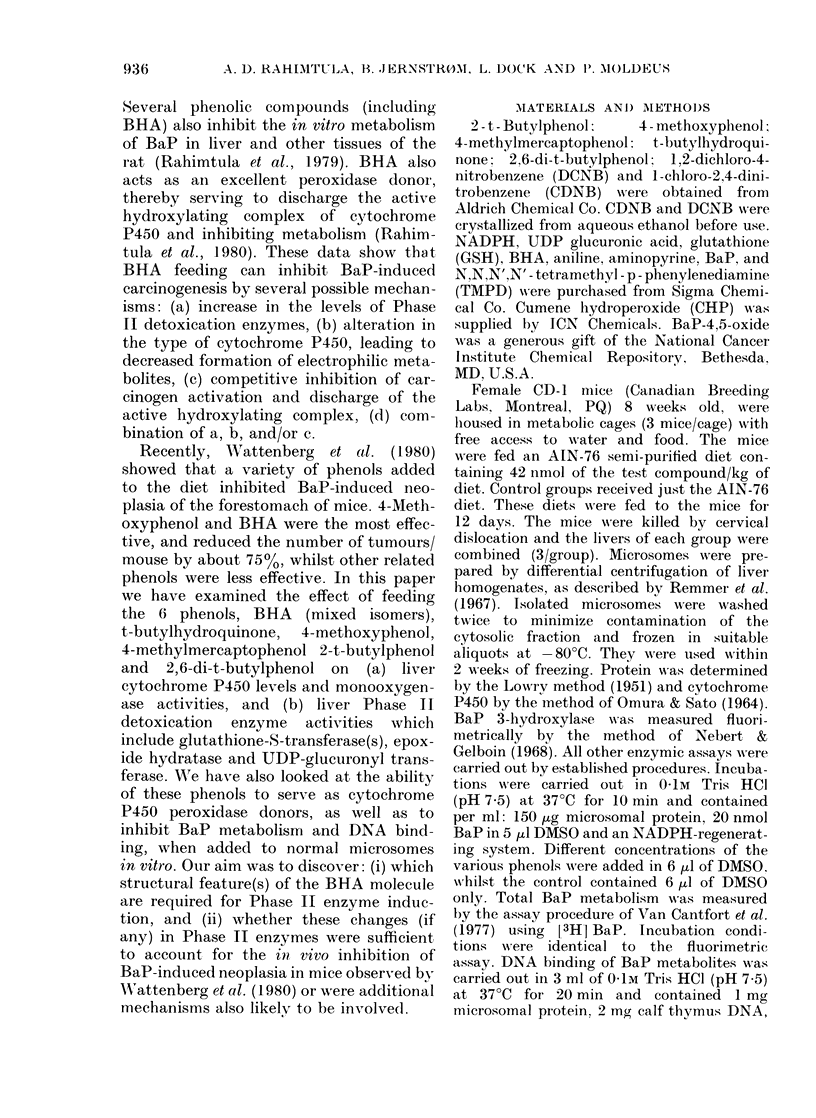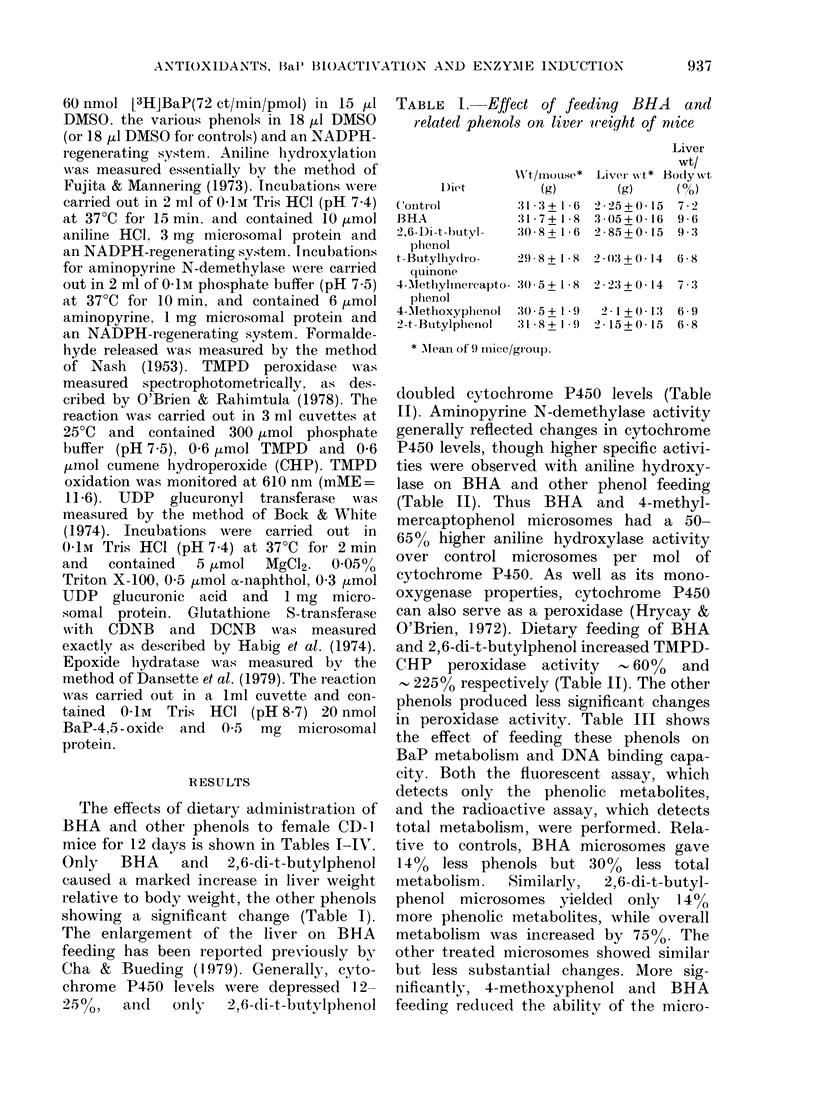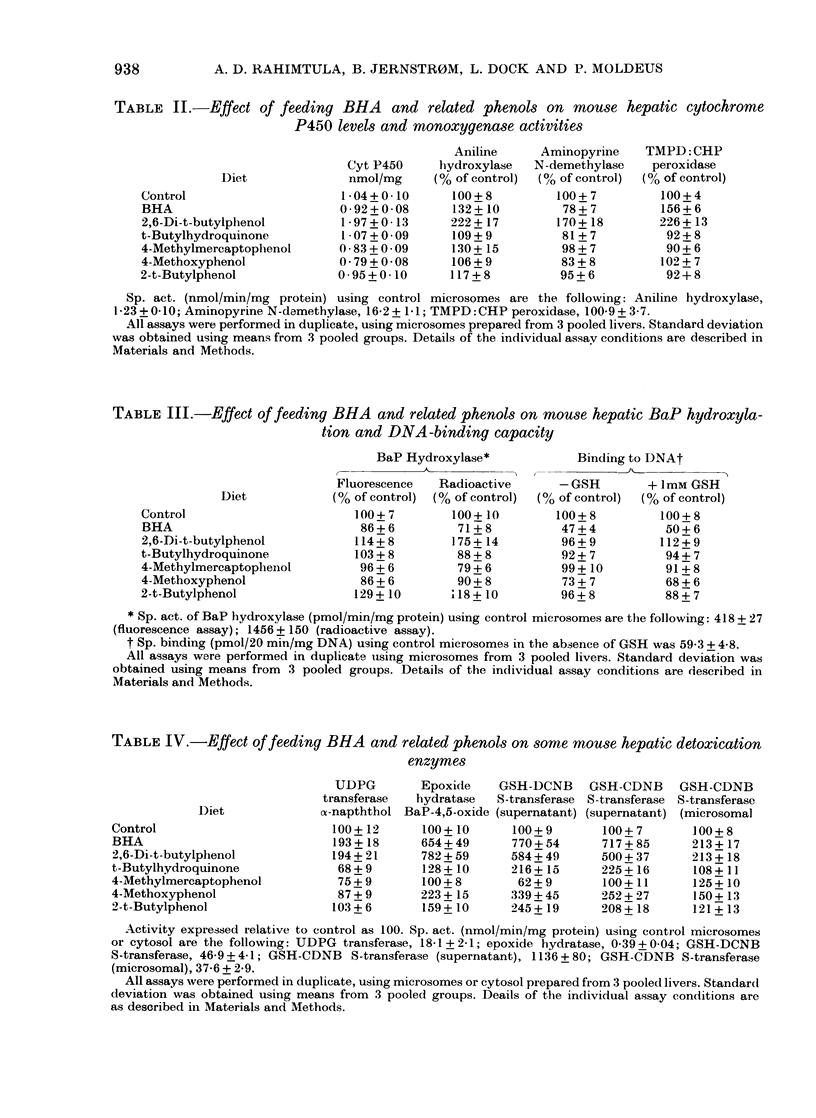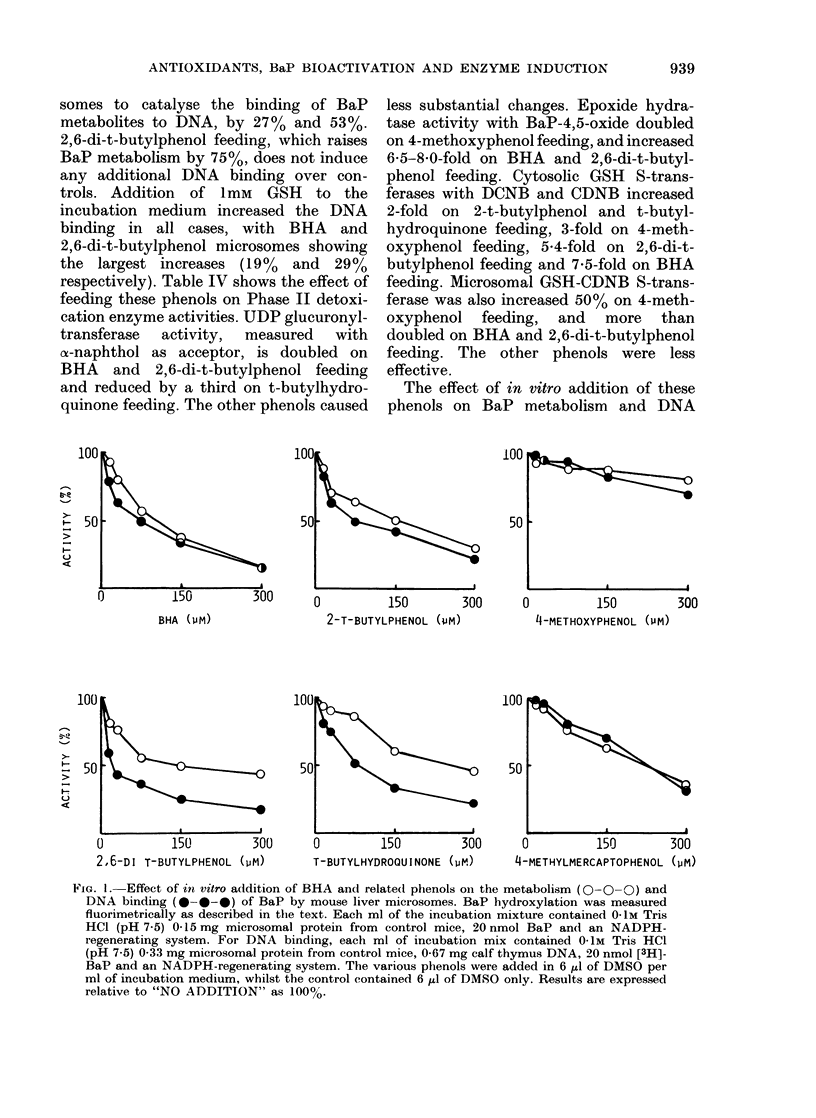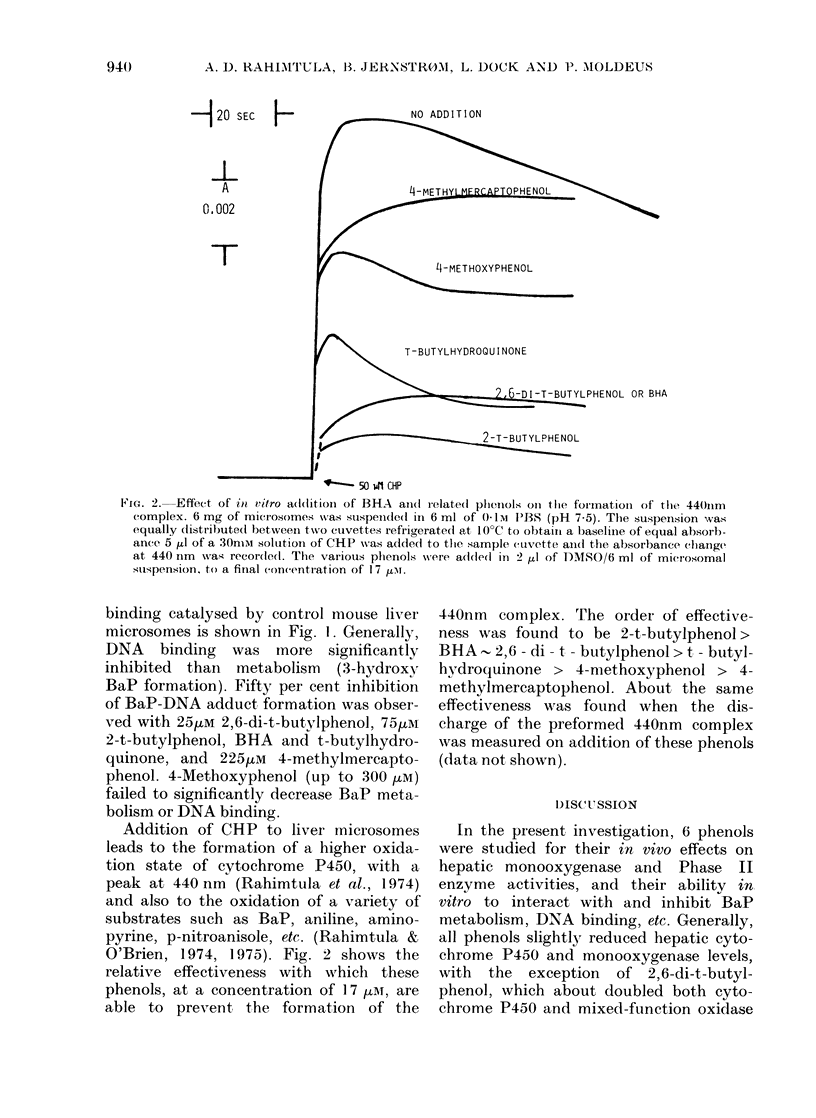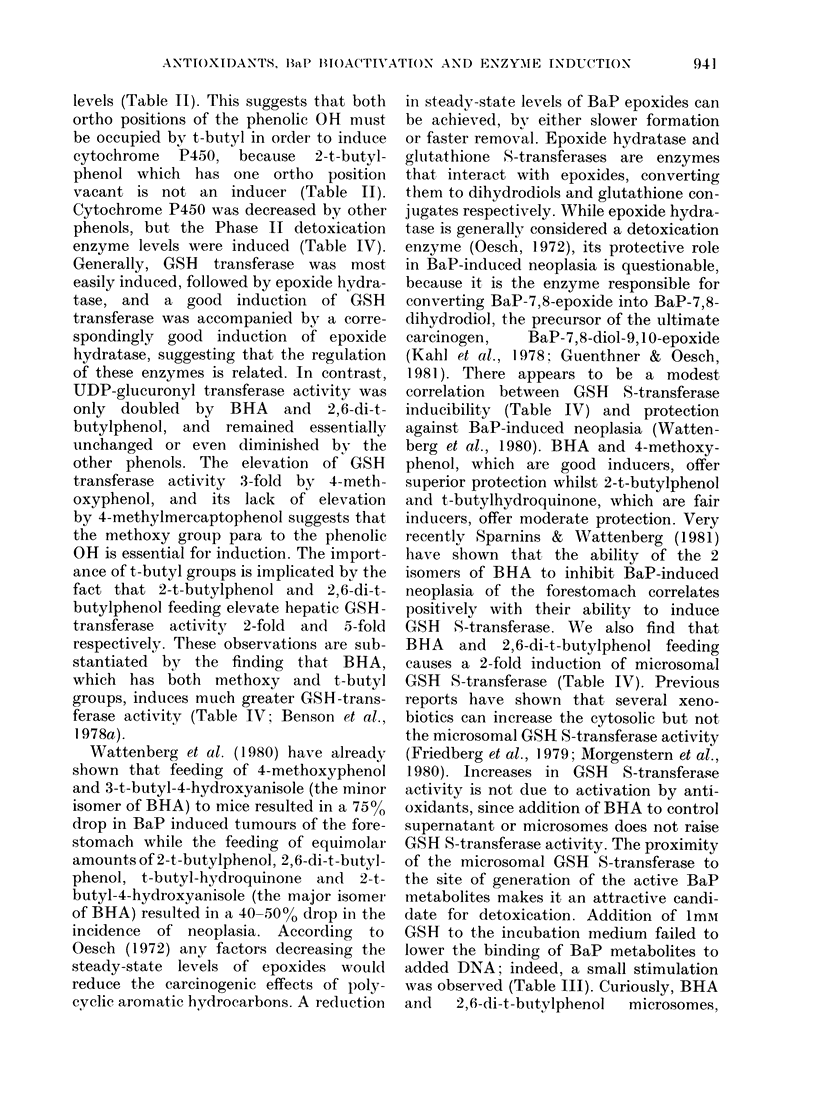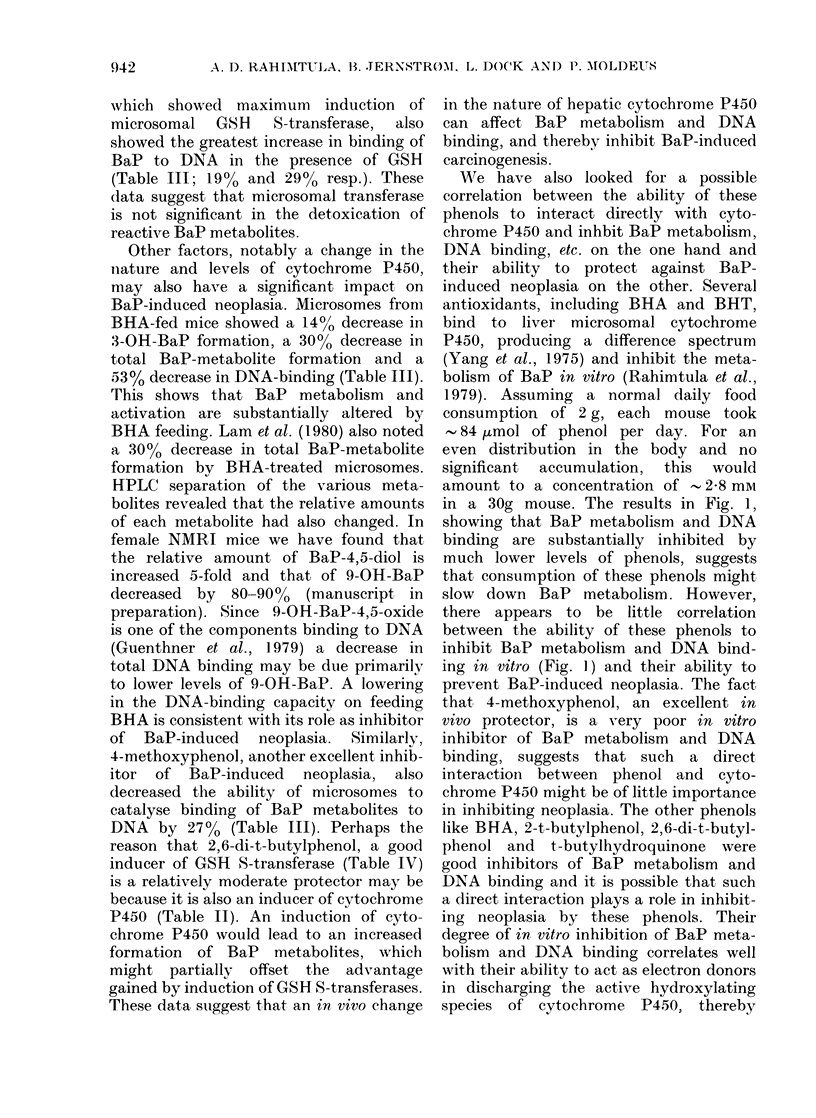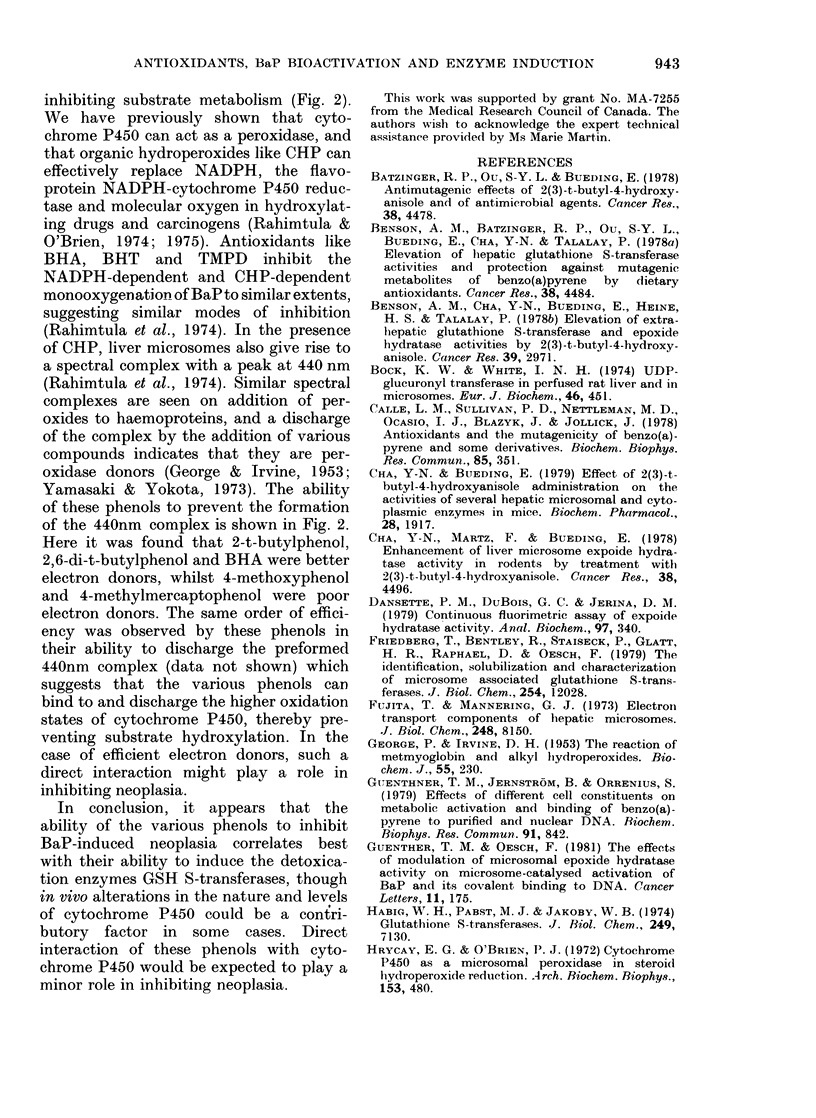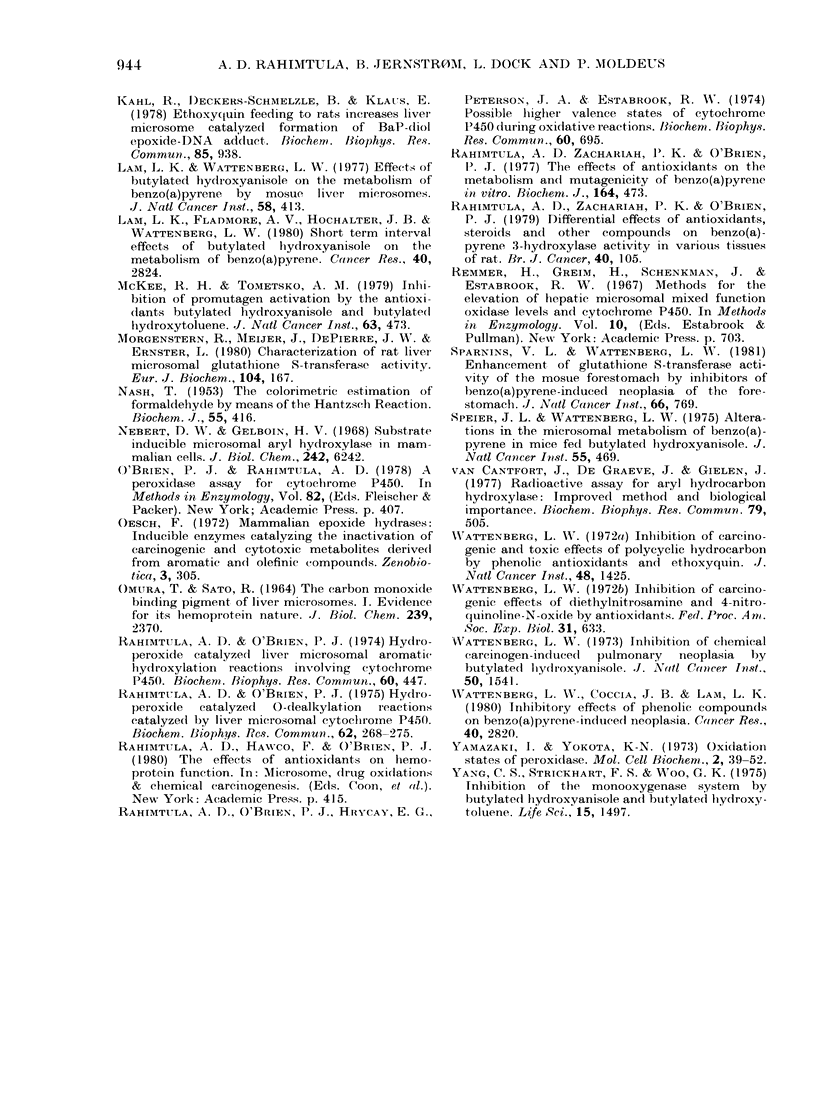# Effects of dietary and in vitro 2(3)-t-butyl-4-hydroxy-anisole and other phenols on hepatic enzyme activities in mice.

**DOI:** 10.1038/bjc.1982.146

**Published:** 1982-06

**Authors:** A. D. Rahimtula, B. Jernström, L. Dock, P. Moldeus

## Abstract

Six phenols [2(3)-t-butyl-4-hydroxyanisole (BHA), 2-t-butylphenol, 4-methoxyphenol, 4-methylmercaptophenol, t-butylhydroquinone and 2,6-di-t-butylphenol] previously shown to be inhibitors of benzo(a)pyrene-induced neoplasia, were examined for their ability to induce in vivo changes in hepatic mono-oxygenase and detoxication enzyme activities, and to act as mono-oxygenase inhibitors when added in vitro. (1) Generally it was found that cytochrome P450 levels were depressed, only 2,6-di-t-butylphenol caused a 2-fold induction (2) Mono-oxygenase activities were significantly altered; BHA and 2,6-di-t-butylphenol caused microsomes to show substantial increases in aniline hydroxylase and peroxidase activities. These microsomes, along with 4-methoxyphenol microsomes, also showed a substantial reduction in DNA binding of benzo(a)pyrene (BaP) metabolites relative to metabolism. (3) Detoxication enzymes glutathione S-transferases and epoxide hydratase were readily induced, the order of effectiveness being: BHA approximately 2,6-di-t-butylphenol greater than 4-methoxyphenol greater than 2-t-butylphenol approximately t-butylhydroquinone (4-methylmercaptophenol failed to induce). (4) In vitro ability to inhibit BaP metabolism and DNA-binding ability was: 2,6-di-t-butylphenol greater than or equal to BHA approximately 2-t-butylphenol greater than t-butylhydroquinone greater than 4-methylmercaptophenol greater than 4-methoxyphenol. (5) Ability in vitro to discharge the activated oxygen complex of cytochrome P450 was: 2,6-di-t-butylphenol approximately 2-t-butylphenol greater than BHA greater t-butylhydroquinone greater than 4-methylmercaptophenol greater than 4-methoxyphenol. The results are consistent with the theory that inhibition of neoplasia is related to inducibility of detoxication enzymes, though alterations in cytochrome P450 could play a significant role in some cases.


Br. J. Cancer (1982) **45**, 935

### EFFECTS OF DIETARY AND *IN VITRO* 2(3)-t-BUTYL-4-HYDROXY-ANISOLE AND OTHER PHENOLS ON HEPATIC ENZYME ACTIVITIES IN MICE

A. D. RAHIMTULA, B. JERNSTRÖM*, L. DOCK* AND P. MOLDEUS*

*From the Department of Biochemistry, Memorial University of Newfoundland, St John's, Newfoundland, Canada A1B 3X9*

Received 4 September 1981 Accepted 10 February 1982

**Summary.—Six phenols [2(3)-t-butyl-4-hydroxyanisole (BHA), 2-t-butylphenol, 4-methoxyphenol, 4-methylmercaptophenol, t-butylhydroquinone and 2,6-di-t-butylphenol] previously shown to be inhibitors of benzo(a)pyrene-induced neoplasia, were examined for their ability to induce *in vivo* changes in hepatic mono-oxygenase and detoxication enzyme activities, and to act as mono-oxygenase inhibitors when added *in vitro*. (1) Generally it was found that cytochrome P450 levels were depressed, only 2,6-di-t-butylphenol caused a 2-fold induction (2) Mono-oxygenase activities were significantly altered; BHA and 2,6-di-t-butylphenol caused microsomes to show substantial increases in aniline hydroxylase and peroxidase activities. These microsomes, along with 4-methoxyphenol microsomes, also showed a substantial reduction in DNA binding of benzo(a)pyrene (BaP) metabolites relative to metabolism. (3) Detoxication enzymes glutathione S-transferases and epoxide hydratase were readily induced, the order of effectiveness being: BHA ~ 2,6-di-t-butylphenol > 4-methoxyphenol > 2-t-butylphenol ~ t-butylhydroquinone (4-methylmercaptophenol failed to induce). (4) *In vitro* ability to inhibit BaP metabolism and DNA-binding ability was: 2,6-di-t-butylphenol ⩾ BHA ~ 2-t-butylphenol > t-butylhydroquinone > 4-methylmercaptophenol > 4-methoxyphenol. (5) Ability *in vitro* to discharge the activated oxygen complex of cytochrome P450 was: 2,6-di-t-butylphenol ~ 2-t-butylphenol > BHA > t-butlyhydroquinone > 4-methylmercaptophenol > 4-methoxyphenol. The results are consistent with the theory that inhibition of neoplasia is related to inducibility of detoxication enzymes, though alterations in cytochrome P450 could play a significant role in some cases.**

THE PHENOLIC ANTIOXIDANT 2(3)-t-butyl-4-hydroxyanisole (BHA) is a widely used food additive which has a number of interesting and potentially important pharmacological properties. When added to commercial diets, it effectively inhibited neoplasia at several sites induced by a variety of carcinogens (Wattenberg, 1972*a*, *b*; 1973). BHA was also found to lower the mutagenicity of BaP and other promutagens both *in vivo* (Batzinger *et al.*, 1978) and *in vitro* (Rahimtula *et al.*, 1977; Calle *et al.*, 1978; McKee & Tometsko, 1979). Dietary administration of BHA to female mice has been shown to increase several fold the specific activities of the Phase II detoxication enzymes, glutathione S-transferases and epoxide hydratase, in the liver and other tissues (Benson *et al.*, 1978*a*, *b*; Cha *et al.*, 1978). UDP-glucuronyl transferase activity was also enhanced (Cha & Bueding, 1979). BHA feeding slightly lowers hepatic cytochrome P450 levels, alters the metabolite profile of BaP and shows a 50% decrease in the *in vitro* binding of BaP metabolites to DNA (Speier & Wattenberg, 1975; Lam & Wattenberg, 1977; Lam *et al.*, 1980).

* Present address: Department of Forensic Medicine, Karolinska Institute, S-104 01 Stockholm 60, Sweden.

Several phenolic compounds (including BHA) also inhibit the *in vitro* metabolism of BaP in liver and other tissues of the rat (Rahimtula *et al.*, 1979). BHA also acts as an excellent peroxidase donor, thereby serving to discharge the active hydroxylating complex of cytochrome P450 and inhibiting metabolism (Rahimtula *et al.*, 1980). These data show that BHA feeding can inhibit BaP-induced carcinogenesis by several possible mechanisms: (a) increase in the levels of Phase II detoxication enzymes, (b) alteration in the type of cytochrome P450, leading to decreased formation of electrophilic metabolites, (c) competitive inhibition of carcinogen activation and discharge of the active hydroxylating complex, (d) combination of a, b, and/or c.

Recently, Wattenberg *et al.* (1980) showed that a variety of phenols added to the diet inhibited BaP-induced neoplasia of the forestomach of mice. 4-Methoxyphenol and BHA were the most effective, and reduced the number of tumours/mouse by about 75%, whilst other related phenols were less effective. In this paper we have examined the effect of feeding the 6 phenols, BHA (mixed isomers), t-butylhydroquinone, 4-methoxyphenol, 4-methylmercaptophenol 2-t-butylphenol and 2,6-di-t-butylphenol on (a) liver cytochrome P450 levels and monooxygenase activities, and (b) liver Phase II detoxication enzyme activities which include glutathione-S-transferase(s), epoxide hydratase and UDP-glucuronyl transferase. We have also looked at the ability of these phenols to serve as cytochrome P450 peroxidase donors, as well as to inhibit BaP metabolism and DNA binding, when added to normal microsomes *in vitro*. Our aim was to discover: (i) which structural feature(s) of the BHA molecule are required for Phase II enzyme induction, and (ii) whether these changes (if any) in Phase II enzymes were sufficient to account for the *in vivo* inhibition of BaP-induced neoplasia in mice observed by Wattenberg *et al.* (1980) or were additional mechanisms also likely to be involved.

### MATERIALS AND METHODS

2-t-Butylphenol; 4-methoxyphenol; 4-methylmercaptophenol; t-butylhydroquinone; 2,6-di-t-butylphenol; 1,2-dichloro-4-nitrobenzene (DCNB) and 1-chloro-2,4-dinitrobenzene (CDNB) were obtained from Aldrich Chemical Co. CDNB and DCNB were crystallized from aqueous ethanol before use. NADPH, UDP glucuronic acid, glutathione (GSH), BHA, aniline, aminopyrine, BaP, and N,N,N′,N′-tetramethyl-p-phenylenediamine (TMPD) were purchased from Sigma Chemical Co. Cumene hydroperoxide (CHP) was supplied by ICN Chemicals. BaP-4,5-oxide was a generous gift of the National Cancer Institute Chemical Repository, Bethesda, MD, U.S.A.

Female CD-1 mice (Canadian Breeding Labs, Montreal, PQ) 8 weeks old, were housed in metabolic cages (3 mice/cage) with free access to water and food. The mice were fed an AIN-76 semi-purified diet containing 42 nmol of the test compound/kg of diet. Control groups received just the AIN-76 diet. These diets were fed to the mice for 12 days. The mice were killed by cervical dislocation and the livers of each group were combined (3/group). Microsomes were prepared by differential centrifugation of liver homogenates, as described by Remmer *et al.* (1967). Isolated microsomes were washed twice to minimize contamination of the cytosolic fraction and frozen in suitable aliquots at −80°C. They were used within 2 weeks of freezing. Protein was determined by the Lowry method (1951) and cytochrome P450 by the method of Omura & Sato (1964). BaP 3-hydroxylase was measured fluorimetrically by the method of Nebert & Gelboin (1968). All other enzymic assays were carried out by established procedures. Incubations were carried out in 0·1M Tris HCl (pH 7·5) at 37°C for 10 min and contained per ml: 150 μg microsomal protein, 20 nmol BaP in 5 μl DMSO and an NADPH-regenerating system. Different concentrations of the various phenols were added in 6 μl of DMSO, whilst the control contained 6 μl of DMSO only. Total BaP metabolism was measured by the assay procedure of Van Cantfort *et al.* (1977) using [$^3$H] BaP. Incubation conditions were identical to the fluorimetric assay. DNA binding of BaP metabolites was carried out in 3 ml of 0·1M Tris HCl (pH 7·5) at 37°C for 20 min and contained 1 mg microsomal protein, 2 mg calf thymus DNA,

60 nmol [$^{3}$H]BaP(72 ct/min/pmol) in 15 $\mu$l DMSO, the various phenols in 18 $\mu$l DMSO (or 18 $\mu$l DMSO for controls) and an NADPH-regenerating system. Aniline hydroxylation was measured essentially by the method of Fujita & Mannering (1973). Incubations were carried out in 2 ml of 0·1M Tris HCl (pH 7·4) at 37°C for 15 min, and contained 10 $\mu$mol aniline HCl, 3 mg microsomal protein and an NADPH-regenerating system. Incubations for aminopyrine N-demethylase were carried out in 2 ml of 0·1M phosphate buffer (pH 7·5) at 37°C for 10 min, and contained 6 $\mu$mol aminopyrine, 1 mg microsomal protein and an NADPH-regenerating system. Formaldehyde released was measured by the method of Nash (1953). TMPD peroxidase was measured spectrophotometrically, as described by O'Brien & Rahimtula (1978). The reaction was carried out in 3 ml cuvettes at 25°C and contained 300 $\mu$mol phosphate buffer (pH 7·5), 0·6 $\mu$mol TMPD and 0·6 $\mu$mol cumene hydroperoxide (CHP). TMPD oxidation was monitored at 610 nm (mME = 11·6). UDP glucuronyl transferase was measured by the method of Bock & White (1974). Incubations were carried out in 0·1M Tris HCl (pH 7·4) at 37°C for 2 min and contained 5 $\mu$mol $MgCl_2$, 0·05% Triton X-100, 0·5 $\mu$mol $\alpha$-naphthol, 0·3 $\mu$mol UDP glucuronic acid and 1 mg microsomal protein. Glutathione S-transferase with CDNB and DCNB was measured exactly as described by Habig *et al.* (1974). Epoxide hydratase was measured by the method of Dansette *et al.* (1979). The reaction was carried out in a 1ml cuvette and contained 0·1M Tris HCl (pH 8·7) 20 nmol BaP-4,5-oxide and 0·5 mg microsomal protein.

### RESULTS

The effects of dietary administration of BHA and other phenols to female CD-1 mice for 12 days is shown in Tables I–IV. Only BHA and 2,6-di-t-butylphenol caused a marked increase in liver weight relative to body weight, the other phenols showing a significant change (Table I). The enlargement of the liver on BHA feeding has been reported previously by Cha & Bueding (1979). Generally, cytochrome P450 levels were depressed 12–25%, and only 2,6-di-t-butylphenol doubled cytochrome P450 levels (Table II). Aminopyrine N-demethylase activity generally reflected changes in cytochrome P450 levels, though higher specific activities were observed with aniline hydroxylase on BHA and other phenol feeding (Table II). Thus BHA and 4-methylmercaptophenol microsomes had a 50–65% higher aniline hydroxylase activity over control microsomes per mol of cytochrome P450. As well as its monooxygenase properties, cytochrome P450 can also serve as a peroxidase (Hrycay & O'Brien, 1972). Dietary feeding of BHA and 2,6-di-t-butylphenol increased TMPD-CHP peroxidase activity ~60% and ~225% respectively (Table II). The other phenols produced less significant changes in peroxidase activity. Table III shows the effect of feeding these phenols on BaP metabolism and DNA binding capacity. Both the fluorescent assay, which detects only the phenolic metabolites, and the radioactive assay, which detects total metabolism, were performed. Relative to controls, BHA microsomes gave 14% less phenols but 30% less total metabolism. Similarly, 2,6-di-t-butylphenol microsomes yielded only 14% more phenolic metabolites, while overall metabolism was increased by 75%. The other treated microsomes showed similar but less substantial changes. More significantly, 4-methoxyphenol and BHA feeding reduced the ability of the micro-

TABLE I.—*Effect of feeding BHA and related phenols on liver weight of mice*

| Diet | Wt/mouse* (g) | Liver wt* (g) | Liver wt/ Body wt (%) |
|---|---|---|---|
| Control | 31·3 ± 1·6 | 2·25 ± 0·15 | 7·2 |
| BHA | 31·7 ± 1·8 | 3·05 ± 0·16 | 9·6 |
| 2,6-Di-t-butyl-phenol | 30·8 ± 1·6 | 2·85 ± 0·15 | 9·3 |
| t-Butylhydro-quinone | 29·8 ± 1·8 | 2·03 ± 0·14 | 6·8 |
| 4-Methylmercapto-phenol | 30·5 ± 1·8 | 2·23 ± 0·14 | 7·3 |
| 4-Methoxyphenol | 30·5 ± 1·9 | 2·1 ± 0·13 | 6·9 |
| 2-t-Butylphenol | 31·8 ± 1·9 | 2·15 ± 0·15 | 6·8 |

* Mean of 9 mice/group.

TABLE II.—*Effect of feeding BHA and related phenols on mouse hepatic cytochrome P450 levels and monoxygenase activities*

| Diet | Cyt P450 nmol/mg | Aniline hydroxylase (% of control) | Aminopyrine N-demethylase (% of control) | TMPD:CHP peroxidase (% of control) |
|---|---|---|---|---|
| Control | 1·04 ± 0·10 | 100 ± 8 | 100 ± 7 | 100 ± 4 |
| BHA | 0·92 ± 0·08 | 132 ± 10 | 78 ± 7 | 156 ± 6 |
| 2,6-Di-t-butylphenol | 1·97 ± 0·13 | 222 ± 17 | 170 ± 18 | 226 ± 13 |
| t-Butylhydroquinone | 1·07 ± 0·09 | 109 ± 9 | 81 ± 7 | 92 ± 8 |
| 4-Methylmercaptophenol | 0·83 ± 0·09 | 130 ± 15 | 98 ± 7 | 90 ± 6 |
| 4-Methoxyphenol | 0·79 ± 0·08 | 106 ± 9 | 83 ± 8 | 102 ± 7 |
| 2-t-Butylphenol | 0·95 ± 0·10 | 117 ± 8 | 95 ± 6 | 92 + 8 |

Sp. act. (nmol/min/mg protein) using control microsomes are the following: Aniline hydroxylase, 1·23 ± 0·10; Aminopyrine N-demethylase, 16·2 ± 1·1; TMPD:CHP peroxidase, 100·9 ± 3·7.

All assays were performed in duplicate, using microsomes prepared from 3 pooled livers. Standard deviation was obtained using means from 3 pooled groups. Details of the individual assay conditions are described in Materials and Methods.

TABLE III.—*Effect of feeding BHA and related phenols on mouse hepatic BaP hydroxylation and DNA-binding capacity*

| Diet | BaP Hydroxylase* | | Binding to DNA† | |
|---|---|---|---|---|
| | Fluorescence (% of control) | Radioactive (% of control) | − GSH (% of control) | + 1mM GSH (% of control) |
| Control | 100 ± 7 | 100 ± 10 | 100 ± 8 | 100 ± 8 |
| BHA | 86 ± 6 | 71 ± 8 | 47 ± 4 | 50 ± 6 |
| 2,6-Di-t-butylphenol | 114 ± 8 | 175 ± 14 | 96 ± 9 | 112 ± 9 |
| t-Butylhydroquinone | 103 ± 8 | 88 ± 8 | 92 ± 7 | 94 ± 7 |
| 4-Methylmercaptophenol | 96 ± 6 | 79 ± 6 | 99 ± 10 | 91 ± 8 |
| 4-Methoxyphenol | 86 ± 6 | 90 ± 8 | 73 ± 7 | 68 ± 6 |
| 2-t-Butylphenol | 129 ± 10 | 118 ± 10 | 96 ± 8 | 88 ± 7 |

* Sp. act. of BaP hydroxylase (pmol/min/mg protein) using control microsomes are the following: 418 ± 27 (fluorescence assay); 1456 ± 150 (radioactive assay).

† Sp. binding (pmol/20 min/mg DNA) using control microsomes in the absence of GSH was 59·3 ± 4·8.

All assays were performed in duplicate using microsomes from 3 pooled livers. Standard deviation was obtained using means from 3 pooled groups. Details of the individual assay conditions are described in Materials and Methods.

TABLE IV.—*Effect of feeding BHA and related phenols on some mouse hepatic detoxication enzymes*

| Diet | UDPG transferase α-napththol | Epoxide hydratase BaP-4,5-oxide | GSH-DCNB S-transferase (supernatant) | GSH-CDNB S-transferase (supernatant) | GSH-CDNB S-transferase (microsomal |
|---|---|---|---|---|---|
| Control | 100 ± 12 | 100 ± 10 | 100 ± 9 | 100 ± 7 | 100 ± 8 |
| BHA | 193 ± 18 | 654 ± 49 | 770 ± 54 | 717 ± 85 | 213 ± 17 |
| 2,6-Di-t-butylphenol | 194 ± 21 | 782 ± 59 | 584 ± 49 | 500 ± 37 | 213 ± 18 |
| t-Butylhydroquinone | 68 ± 9 | 128 ± 10 | 216 ± 15 | 225 ± 16 | 108 ± 11 |
| 4-Methylmercaptophenol | 75 ± 9 | 100 ± 8 | 62 ± 9 | 100 ± 11 | 125 ± 10 |
| 4-Methoxyphenol | 87 ± 9 | 223 ± 15 | 339 ± 45 | 252 ± 27 | 150 ± 13 |
| 2-t-Butylphenol | 103 ± 6 | 159 ± 10 | 245 ± 19 | 208 ± 18 | 121 ± 13 |

Activity expressed relative to control as 100. Sp. act. (nmol/min/mg protein) using control microsomes or cytosol are the following: UDPG transferase, 18·1 ± 2·1; epoxide hydratase, 0·39 ± 0·04; GSH-DCNB S-transferase, 46·9 ± 4·1; GSH-CDNB S-transferase (supernatant), 1136 ± 80; GSH-CDNB S-transferase (microsomal), 37·6 ± 2·9.

All assays were performed in duplicate, using microsomes or cytosol prepared from 3 pooled livers. Standard deviation was obtained using means from 3 pooled groups. Deails of the individual assay conditions are as described in Materials and Methods.

somes to catalyse the binding of BaP metabolites to DNA, by 27% and 53%. 2,6-di-t-butylphenol feeding, which raises BaP metabolism by 75%, does not induce any additional DNA binding over controls. Addition of 1mM GSH to the incubation medium increased the DNA binding in all cases, with BHA and 2,6-di-t-butylphenol microsomes showing the largest increases (19% and 29% respectively). Table IV shows the effect of feeding these phenols on Phase II detoxication enzyme activities. UDP glucuronyltransferase activity, measured with α-naphthol as acceptor, is doubled on BHA and 2,6-di-t-butylphenol feeding and reduced by a third on t-butylhydroquinone feeding. The other phenols caused less substantial changes. Epoxide hydratase activity with BaP-4,5-oxide doubled on 4-methoxyphenol feeding, and increased 6·5–8·0-fold on BHA and 2,6-di-t-butylphenol feeding. Cytosolic GSH S-transferases with DCNB and CDNB increased 2-fold on 2-t-butylphenol and t-butylhydroquinone feeding, 3-fold on 4-methoxyphenol feeding, 5·4-fold on 2,6-di-t-butylphenol feeding and 7·5-fold on BHA feeding. Microsomal GSH-CDNB S-transferase was also increased 50% on 4-methoxyphenol feeding, and more than doubled on BHA and 2,6-di-t-butylphenol feeding. The other phenols were less effective.

The effect of *in vitro* addition of these phenols on BaP metabolism and DNA

FIG. 1.—Effect of *in vitro* addition of BHA and related phenols on the metabolism (○–○–○) and DNA binding (●–●–●) of BaP by mouse liver microsomes. BaP hydroxylation was measured fluorimetrically as described in the text. Each ml of the incubation mixture contained 0·1M Tris HCl (pH 7·5) 0·15 mg microsomal protein from control mice, 20 nmol BaP and an NADPH-regenerating system. For DNA binding, each ml of incubation mix contained 0·1M Tris HCl (pH 7·5) 0·33 mg microsomal protein from control mice, 0·67 mg calf thymus DNA, 20 nmol [$^3$H]-BaP and an NADPH-regenerating system. The various phenols were added in 6 μl of DMSO per ml of incubation medium, whilst the control contained 6 μl of DMSO only. Results are expressed relative to "NO ADDITION" as 100%.

FIG. 2.—Effect of *in vitro* addition of BHA and related phenols on the formation of the 440nm complex. 6 mg of microsomes was suspended in 6 ml of 0·1M PBS (pH 7·5). The suspension was equally distributed between two cuvettes refrigerated at 10°C to obtain a baseline of equal absorbance 5 μl of a 30mM solution of CHP was added to the sample cuvette and the absorbance change at 440 nm was recorded. The various phenols were added in 2 μl of DMSO/6 ml of microsomal suspension, to a final concentration of 17 μM.

binding catalysed by control mouse liver microsomes is shown in Fig. 1. Generally, DNA binding was more significantly inhibited than metabolism (3-hydroxy BaP formation). Fifty per cent inhibition of BaP-DNA adduct formation was observed with 25μM 2,6-di-t-butylphenol, 75μM 2-t-butylphenol, BHA and t-butylhydroquinone, and 225μM 4-methylmercaptophenol. 4-Methoxyphenol (up to 300 μM) failed to significantly decrease BaP metabolism or DNA binding.

Addition of CHP to liver microsomes leads to the formation of a higher oxidation state of cytochrome P450, with a peak at 440 nm (Rahimtula *et al.*, 1974) and also to the oxidation of a variety of substrates such as BaP, aniline, aminopyrine, p-nitroanisole, *etc.* (Rahimtula & O'Brien, 1974, 1975). Fig. 2 shows the relative effectiveness with which these phenols, at a concentration of 17 μM, are able to prevent the formation of the 440nm complex. The order of effectiveness was found to be 2-t-butylphenol > BHA ~ 2,6 - di - t - butylphenol > t - butylhydroquinone > 4-methoxyphenol > 4-methylmercaptophenol. About the same effectiveness was found when the discharge of the preformed 440nm complex was measured on addition of these phenols (data not shown).

### DISCUSSION

In the present investigation, 6 phenols were studied for their *in vivo* effects on hepatic monooxygenase and Phase II enzyme activities, and their ability *in vitro* to interact with and inhibit BaP metabolism, DNA binding, *etc.* Generally, all phenols slightly reduced hepatic cytochrome P450 and monooxygenase levels, with the exception of 2,6-di-t-butylphenol, which about doubled both cytochrome P450 and mixed-function oxidase levels (Table II). This suggests that both ortho positions of the phenolic OH must be occupied by t-butyl in order to induce cytochrome P450, because 2-t-butylphenol which has one ortho position vacant is not an inducer (Table II). Cytochrome P450 was decreased by other phenols, but the Phase II detoxication enzyme levels were induced (Table IV). Generally, GSH transferase was most easily induced, followed by epoxide hydratase, and a good induction of GSH transferase was accompanied by a correspondingly good induction of epoxide hydratase, suggesting that the regulation of these enzymes is related. In contrast, UDP-glucuronyl transferase activity was only doubled by BHA and 2,6-di-t-butylphenol, and remained essentially unchanged or even diminished by the other phenols. The elevation of GSH transferase activity 3-fold by 4-methoxyphenol, and its lack of elevation by 4-methylmercaptophenol suggests that the methoxy group para to the phenolic OH is essential for induction. The importance of t-butyl groups is implicated by the fact that 2-t-butylphenol and 2,6-di-t-butylphenol feeding elevate hepatic GSH-transferase activity 2-fold and 5-fold respectively. These observations are substantiated by the finding that BHA, which has both methoxy and t-butyl groups, induces much greater GSH-transferase activity (Table IV; Benson *et al.*, 1978*a*).

Wattenberg *et al.* (1980) have already shown that feeding of 4-methoxyphenol and 3-t-butyl-4-hydroxyanisole (the minor isomer of BHA) to mice resulted in a 75% drop in BaP induced tumours of the forestomach while the feeding of equimolar amounts of 2-t-butylphenol, 2,6-di-t-butylphenol, t-butyl-hydroquinone and 2-t-butyl-4-hydroxyanisole (the major isomer of BHA) resulted in a 40–50% drop in the incidence of neoplasia. According to Oesch (1972) any factors decreasing the steady-state levels of epoxides would reduce the carcinogenic effects of polycyclic aromatic hydrocarbons. A reduction in steady-state levels of BaP epoxides can be achieved, by either slower formation or faster removal. Epoxide hydratase and glutathione S-transferases are enzymes that interact with epoxides, converting them to dihydrodiols and glutathione conjugates respectively. While epoxide hydratase is generally considered a detoxication enzyme (Oesch, 1972), its protective role in BaP-induced neoplasia is questionable, because it is the enzyme responsible for converting BaP-7,8-epoxide into BaP-7,8-dihydrodiol, the precursor of the ultimate carcinogen, BaP-7,8-diol-9,10-epoxide (Kahl *et al.*, 1978; Guenthner & Oesch, 1981). There appears to be a modest correlation between GSH S-transferase inducibility (Table IV) and protection against BaP-induced neoplasia (Wattenberg *et al.*, 1980). BHA and 4-methoxyphenol, which are good inducers, offer superior protection whilst 2-t-butylphenol and t-butylhydroquinone, which are fair inducers, offer moderate protection. Very recently Sparnins & Wattenberg (1981) have shown that the ability of the 2 isomers of BHA to inhibit BaP-induced neoplasia of the forestomach correlates positively with their ability to induce GSH S-transferase. We also find that BHA and 2,6-di-t-butylphenol feeding causes a 2-fold induction of microsomal GSH S-transferase (Table IV). Previous reports have shown that several xenobiotics can increase the cytosolic but not the microsomal GSH S-transferase activity (Friedberg *et al.*, 1979; Morgenstern *et al.*, 1980). Increases in GSH S-transferase activity is not due to activation by antioxidants, since addition of BHA to control supernatant or microsomes does not raise GSH S-transferase activity. The proximity of the microsomal GSH S-transferase to the site of generation of the active BaP metabolites makes it an attractive candidate for detoxication. Addition of 1mM GSH to the incubation medium failed to lower the binding of BaP metabolites to added DNA; indeed, a small stimulation was observed (Table III). Curiously, BHA and 2,6-di-t-butylphenol microsomes, which showed maximum induction of microsomal GSH S-transferase, also showed the greatest increase in binding of BaP to DNA in the presence of GSH (Table III; 19% and 29% resp.). These data suggest that microsomal transferase is not significant in the detoxication of reactive BaP metabolites.

Other factors, notably a change in the nature and levels of cytochrome P450, may also have a significant impact on BaP-induced neoplasia. Microsomes from BHA-fed mice showed a 14% decrease in 3-OH-BaP formation, a 30% decrease in total BaP-metabolite formation and a 53% decrease in DNA-binding (Table III). This shows that BaP metabolism and activation are substantially altered by BHA feeding. Lam *et al.* (1980) also noted a 30% decrease in total BaP-metabolite formation by BHA-treated microsomes. HPLC separation of the various metabolites revealed that the relative amounts of each metabolite had also changed. In female NMRI mice we have found that the relative amount of BaP-4,5-diol is increased 5-fold and that of 9-OH-BaP decreased by 80–90% (manuscript in preparation). Since 9-OH-BaP-4,5-oxide is one of the components binding to DNA (Guenthner *et al.*, 1979) a decrease in total DNA binding may be due primarily to lower levels of 9-OH-BaP. A lowering in the DNA-binding capacity on feeding BHA is consistent with its role as inhibitor of BaP-induced neoplasia. Similarly, 4-methoxyphenol, another excellent inhibitor of BaP-induced neoplasia, also decreased the ability of microsomes to catalyse binding of BaP metabolites to DNA by 27% (Table III). Perhaps the reason that 2,6-di-t-butylphenol, a good inducer of GSH S-transferase (Table IV) is a relatively moderate protector may be because it is also an inducer of cytochrome P450 (Table II). An induction of cytochrome P450 would lead to an increased formation of BaP metabolites, which might partially offset the advantage gained by induction of GSH S-transferases. These data suggest that an *in vivo* change in the nature of hepatic cytochrome P450 can affect BaP metabolism and DNA binding, and thereby inhibit BaP-induced carcinogenesis.

We have also looked for a possible correlation between the ability of these phenols to interact directly with cytochrome P450 and inhbit BaP metabolism, DNA binding, *etc.* on the one hand and their ability to protect against BaP-induced neoplasia on the other. Several antioxidants, including BHA and BHT, bind to liver microsomal cytochrome P450, producing a difference spectrum (Yang *et al.*, 1975) and inhibit the metabolism of BaP *in vitro* (Rahimtula *et al.*, 1979). Assuming a normal daily food consumption of 2 g, each mouse took ~84 μmol of phenol per day. For an even distribution in the body and no significant accumulation, this would amount to a concentration of ~2·8 mM in a 30g mouse. The results in Fig. 1, showing that BaP metabolism and DNA binding are substantially inhibited by much lower levels of phenols, suggests that consumption of these phenols might slow down BaP metabolism. However, there appears to be little correlation between the ability of these phenols to inhibit BaP metabolism and DNA binding *in vitro* (Fig. 1) and their ability to prevent BaP-induced neoplasia. The fact that 4-methoxyphenol, an excellent *in vivo* protector, is a very poor *in vitro* inhibitor of BaP metabolism and DNA binding, suggests that such a direct interaction between phenol and cytochrome P450 might be of little importance in inhibiting neoplasia. The other phenols like BHA, 2-t-butylphenol, 2,6-di-t-butylphenol and t-butylhydroquinone were good inhibitors of BaP metabolism and DNA binding and it is possible that such a direct interaction plays a role in inhibiting neoplasia by these phenols. Their degree of *in vitro* inhibition of BaP metabolism and DNA binding correlates well with their ability to act as electron donors in discharging the active hydroxylating species of cytochrome P450, thereby inhibiting substrate metabolism (Fig. 2). We have previously shown that cytochrome P450 can act as a peroxidase, and that organic hydroperoxides like CHP can effectively replace NADPH, the flavoprotein NADPH-cytochrome P450 reductase and molecular oxygen in hydroxylating drugs and carcinogens (Rahimtula & O'Brien, 1974; 1975). Antioxidants like BHA, BHT and TMPD inhibit the NADPH-dependent and CHP-dependent monooxygenation of BaP to similar extents, suggesting similar modes of inhibition (Rahimtula *et al.*, 1974). In the presence of CHP, liver microsomes also give rise to a spectral complex with a peak at 440 nm (Rahimtula *et al.*, 1974). Similar spectral complexes are seen on addition of peroxides to haemoproteins, and a discharge of the complex by the addition of various compounds indicates that they are peroxidase donors (George & Irvine, 1953; Yamasaki & Yokota, 1973). The ability of these phenols to prevent the formation of the 440nm complex is shown in Fig. 2. Here it was found that 2-t-butylphenol, 2,6-di-t-butylphenol and BHA were better electron donors, whilst 4-methoxyphenol and 4-methylmercaptophenol were poor electron donors. The same order of efficiency was observed by these phenols in their ability to discharge the preformed 440nm complex (data not shown) which suggests that the various phenols can bind to and discharge the higher oxidation states of cytochrome P450, thereby preventing substrate hydroxylation. In the case of efficient electron donors, such a direct interaction might play a role in inhibiting neoplasia.

In conclusion, it appears that the ability of the various phenols to inhibit BaP-induced neoplasia correlates best with their ability to induce the detoxication enzymes GSH S-transferases, though *in vivo* alterations in the nature and levels of cytochrome P450 could be a contributory factor in some cases. Direct interaction of these phenols with cytochrome P450 would be expected to play a minor role in inhibiting neoplasia.

This work was supported by grant No. MA-7255 from the Medical Research Council of Canada. The authors wish to acknowledge the expert technical assistance provided by Ms Marie Martin.

### REFERENCES

Batzinger, R. P., Ou, S-Y. L. & Bueding, E. (1978) Antimutagenic effects of 2(3)-t-butyl-4-hydroxyanisole and of antimicrobial agents. *Cancer Res.*, **38**, 4478.

Benson, A. M., Batzinger, R. P., Ou, S-Y. L., Bueding, E., Cha, Y-N. & Talalay, P. (1978*a*) Elevation of hepatic glutathione S-transferase activities and protection against mutagenic metabolites of benzo(a)pyrene by dietary antioxidants. *Cancer Res.*, **38**, 4484.

Benson, A. M., Cha, Y-N., Bueding, E., Heine, H. S. & Talalay, P. (1978*b*) Elevation of extrahepatic glutathione S-transferase and epoxide hydratase activities by 2(3)-t-butyl-4-hydroxyanisole. *Cancer Res.* **39**, 2971.

Bock, K. W. & White, I. N. H. (1974) UDP-glucuronyl transferase in perfused rat liver and in microsomes. *Eur. J. Biochem.*, **46**, 451.

Calle, L. M., Sullivan, P. D., Nettleman, M. D., Ocasio, I. J., Blazyk, J. & Jollick, J. (1978) Antioxidants and the mutagenicity of benzo(a)pyrene and some derivatives. *Biochem. Biophys. Res. Commun.*, **85**, 351.

Cha, Y-N. & Bueding, E. (1979) Effect of 2(3)-t-butyl-4-hydroxyanisole administration on the activities of several hepatic microsomal and cytoplasmic enzymes in mice. *Biochem. Pharmacol.*, **28**, 1917.

Cha, Y-N., Martz, F. & Bueding, E. (1978) Enhancement of liver microsome expoide hydratase activity in rodents by treatment with 2(3)-t-butyl-4-hydroxyanisole. *Cancer Res.*, **38**, 4496.

Dansette, P. M., DuBois, G. C. & Jerina, D. M. (1979) Continuous fluorimetric assay of expoide hydratase activity. *Anal. Biochem.*, **97**, 340.

Friedberg, T., Bentley, R., Staiseck, P., Glatt, H. R., Raphael, D. & Oesch, F. (1979) The identification, solubilization and characterization of microsome associated glutathione S-transferases. *J. Biol. Chem.*, **254**, 12028.

Fujita, T. & Mannering, G. J. (1973) Electron transport components of hepatic microsomes. *J. Biol. Chem.*, **248**, 8150.

George, P. & Irvine, D. H. (1953) The reaction of metmyoglobin and alkyl hydroperoxides. *Biochem. J.*, **55**, 230.

Guenthner, T. M., Jernström, B. & Orrenius, S. (1979) Effects of different cell constituents on metabolic activation and binding of benzo(a)-pyrene to purified and nuclear DNA. *Biochem. Biophys. Res. Commun.* **91**, 842.

Guenther, T. M. & Oesch, F. (1981) The effects of modulation of microsomal epoxide hydratase activity on microsome-catalysed activation of BaP and its covalent binding to DNA. *Cancer Letters*, **11**, 175.

Habig, W. H., Pabst, M. J. & Jakoby, W. B. (1974) Glutathione S-transferases. *J. Biol. Chem.*, **249**, 7130.

Hrycay, E. G. & O'Brien, P. J. (1972) Cytochrome P450 as a microsomal peroxidase in steroid hydroperoxide reduction. *Arch. Biochem. Biophys.*, **153**, 480.

KAHL, R., DECKERS-SCHMELZLE, B. & KLAUS, E. (1978) Ethoxyquin feeding to rats increases liver microsome catalyzed formation of BaP-diol epoxide-DNA adduct. *Biochem. Biophys. Res. Commun.*, **85**, 938.

LAM, L. K. & WATTENBERG, L. W. (1977) Effects of butylated hydroxyanisole on the metabolism of benzo(a)pyrene by mosue liver microsomes. *J. Natl Cancer Inst.*, **58**, 413.

LAM, L. K., FLADMORE, A. V., HOCHALTER, J. B. & WATTENBERG, L. W. (1980) Short term interval effects of butylated hydroxyanisole on the metabolism of benzo(a)pyrene. *Cancer Res.*, **40**, 2824.

MCKEE, R. H. & TOMETSKO, A. M. (1979) Inhibition of promutagen activation by the antioxidants butylated hydroxyanisole and butylated hydroxytoluene. *J. Natl Cancer Inst.*, **63**, 473.

MORGENSTERN, R., MEIJER, J., DEPIERRE, J. W. & ERNSTER, L. (1980) Characterization of rat liver microsomal glutathione S-transferase activity. *Eur. J. Biochem.*, **104**, 167.

NASH, T. (1953) The colorimetric estimation of formaldehyde by means of the Hantzsch Reaction. *Biochem. J.*, **55**, 416.

NEBERT, D. W. & GELBOIN, H. V. (1968) Substrate inducible microsomal aryl hydroxylase in mammalian cells. *J. Biol. Chem.*, **242**, 6242.

O'BRIEN, P. J. & RAHIMTULA, A. D. (1978) A peroxidase assay for cytochrome P450. In *Methods in Enzymology*, Vol. **82**, (Eds. Fleischer & Packer). New York; Academic Press. p. 407.

OESCH, F. (1972) Mammalian epoxide hydrases: Inducible enzymes catalyzing the inactivation of carcinogenic and cytotoxic metabolites derived from aromatic and olefinic compounds. *Zenobiotica*, **3**, 305.

OMURA, T. & SATO, R. (1964) The carbon monoxide binding pigment of liver microsomes. I. Evidence for its hemoprotein nature. *J. Biol. Chem.* **239**, 2370.

RAHIMTULA, A. D. & O'BRIEN, P. J. (1974) Hydroperoxide catalyzed liver microsomal aromatic hydroxylation reactions involving cytochrome P450. *Biochem. Biophys. Res. Commun.*, **60**, 447.

RAHIMTULA, A. D. & O'BRIEN, P. J. (1975) Hydroperoxide catalyzed O-dealkylation reactions catalyzed by liver microsomal cytochrome P450. *Biochem. Biophys. Res. Commun.*, **62**, 268–275.

RAHIMTULA, A. D., HAWCO, F. & O'BRIEN, P. J. (1980) The effects of antioxidants on hemoprotein function. In: Microsome, drug oxidations & chemical carcinogenesis. (Eds. Coon, *et al.*). New York: Academic Press. p. 415.

RAHIMTULA, A. D., O'BRIEN, P. J., HRYCAY, E. G., PETERSON, J. A. & ESTABROOK, R. W. (1974) Possible higher valence states of cytochrome P450 during oxidative reactions. *Biochem. Biophys. Res. Commun.*, **60**, 695.

RAHIMTULA, A. D. ZACHARIAH, P. K. & O'BRIEN, P. J. (1977) The effects of antioxidants on the metabolism and mutagenicity of benzo(a)pyrene *in vitro*. *Biochem. J.*, **164**, 473.

RAHIMTULA, A. D., ZACHARIAH, P. K. & O'BRIEN, P. J. (1979) Differential effects of antioxidants, steroids and other compounds on benzo(a)-pyrene 3-hydroxylase activity in various tissues of rat. *Br. J. Cancer*, **40**, 105.

REMMER, H., GREIM, H., SCHENKMAN, J. & ESTABROOK, R. W. (1967) Methods for the elevation of hepatic microsomal mixed function oxidase levels and cytochrome P450. In *Methods in Enzymology*. Vol. **10**, (Eds. Estabrook & Pullman). New York: Academic Press. p. 703.

SPARNINS, V. L. & WATTENBERG, L. W. (1981) Enhancement of glutathione S-transferase activity of the mosue forestomach by inhibitors of benzo(a)pyrene-induced neoplasia of the forestomach. *J. Natl Cancer Inst.*, **66**, 769.

SPEIER, J. L. & WATTENBERG, L. W. (1975) Alterations in the microsomal metabolism of benzo(a)-pyrene in mice fed butylated hydroxyanisole. *J. Natl Cancer Inst.* **55**, 469.

VAN CANTFORT, J., DE GRAEVE, J. & GIELEN, J. (1977) Radioactive assay for aryl hydrocarbon hydroxylase: Improved method and biological importance. *Biochem. Biophys. Res. Commun.* **79**, 505.

WATTENBERG, L. W. (1972*a*) Inhibition of carcinogenic and toxic effects of polycyclic hydrocarbon by phenolic antioxidants and ethoxyquin. *J. Natl Cancer Inst.*, **48**, 1425.

WATTENBERG, L. W. (1972*b*) Inhibition of carcinogenic effects of diethylnitrosamine and 4-nitroquinoline-N-oxide by antioxidants. *Fed. Proc. Am. Soc. Exp. Biol.* **31**, 633.

WATTENBERG, L. W. (1973) Inhibition of chemical carcinogen-induced pulmonary neoplasia by butylated hydroxyanisole. *J. Natl Cancer Inst.*, **50**, 1541.

WATTENBERG, L. W., COCCIA, J. B. & LAM, L. K. (1980) Inhibitory effects of phenolic compounds on benzo(a)pyrene-induced neoplasia. *Cancer Res.*, **40**, 2820.

YAMAZAKI, I. & YOKOTA, K-N. (1973) Oxidation states of peroxidase. *Mol. Cell Biochem.*, **2**, 39–52.

YANG, C. S., STRICKHART, F. S. & WOO, G. K. (1975) Inhibition of the monooxygenase system by butylated hydroxyanisole and butylated hydroxytoluene. *Life Sci.*, **15**, 1497.